# Code Social: Integrating SDoH into Emergency Resident Education

**DOI:** 10.21980/J8.52163

**Published:** 2025-10-31

**Authors:** Layla Abubshait, Sandra Guirguis, Savannah Pocquette, Timothy Cofer, Angelina Birrell-Lopez

**Affiliations:** * Jefferson Einstein Montgomery Hospital, Department of Emergency Medicine East Norriton, PA; ^ Sidney Kimmel Medical College, Thomas Jefferson University, Philadelphia, PA

## Abstract

**Audience:**

This small group curriculum is geared toward medical students and residents of all levels of training in emergency medicine programs.

**Introduction:**

Emergency departments serve as critical first points of contact for patients with acute health needs, often exacerbated by social determinants of health (SDoH). Incorporating SDoH awareness into emergency medicine practice is essential for addressing immediate health concerns, preventing future crises, and reducing the burden on healthcare systems. By identifying and addressing social factors, emergency providers can break cycles of recurrent emergencies, connect patients with community resources, and foster trust-based, patient-centered relationships. Training emergency medicine residents in SDoH is crucial for preparing them to provide comprehensive, equitable care to diverse populations, enhancing their ability to advocate for vulnerable groups and reduce health disparities. This simulation curriculum aims to immerse residents in scenarios that highlight SDoH impacts, equipping them with the skills to recognize, address, and provide resources for patients affected by social determinants, ultimately improving overall patient outcomes and quality of care in emergency settings.

**Educational Objectives:**

The educational objectives of this activity are to:

1. Enhance residents’ understanding and recognition of (SDoH) in emergency medicine.
2. Develop residents’ skills in addressing SDoH within the emergency medicine setting.
3. Foster a patient-centered approach that promotes health equity in emergency medicine.
4. Identify local resources available to address various SDOH.

**Educational Methods:**

The curriculum employs a comprehensive, multi-faceted approach to educate emergency medicine residents about SDoH. It combines didactic lectures, online simulations, clinical case scenarios, debriefing sessions, and community engagement activities to provide a well-rounded learning experience.

**Research Methods:**

Pre- and post-curriculum surveys were employed to evaluate the effectiveness of the SDoH training program for residents. These surveys assessed residents’ confidence in understanding SDoH, their ability to recognize SDoH impacts on emergency department patients, and their knowledge of related community resources and support services, before and after the curriculum. The pre-survey established a baseline, while the post-survey measured the impact of the training. Additionally, verbal feedback from residents and faculty was collected to gather insights and suggestions for improvement. This comprehensive evaluation approach aimed to ensure the curriculum’s responsiveness to residents’ needs and facilitate ongoing refinement of the simulation program.

**Results:**

A comprehensive survey of 16 emergency medicine residents across various training levels achieved full participation, assessing their understanding of SDoH, recognition of SDoH impacts on patients, and knowledge of relevant community resources. Analysis of pre- and post-curriculum responses demonstrated that while residents had initial SDoH awareness, the curriculum significantly deepened their comprehension and practical application of SDoH concepts. Notably, residents showed substantial improvement in identifying SDoH effects on patients’ overall health and well-being, with the most significant progress in their familiarity with SDoH-related community resources. These results highlight the curriculum’s success in equipping emergency medicine residents to provide holistic, equitable care and champion health equity in their practice.

**Discussion:**

The innovative SDoH curriculum for emergency medicine residents shows promising results in enhancing comprehensive and equitable care delivery. Utilizing diverse educational methods including lectures, simulations, case studies, and community engagement, the program effectively translates theoretical SDoH knowledge into practical skills. Significant improvements in residents’ understanding, identification, and knowledge of local SDoH resources highlight the curriculum’s efficacy. These outcomes indicate that the program successfully prepares residents to navigate the complex relationship between social factors and health outcomes in emergency settings, potentially fostering more patient-centered care and advancing health equity. While these initial findings are encouraging, it’s important to recognize the study’s limitations and consider future research opportunities to further validate and refine the curriculum’s impact.

**Topics:**

Social medicine, social determinants of health, homelessness, health literacy, limited English proficiency.

## USER GUIDE

**Table:** 

List of Resources:	
Abstract	87
User Guide	89
Small Groups Learning Materials	92
Appendix A: Part One: Case-Based Small Group Sessions	92
Appendix B: Part Two: Community Resource Panel Discussion	111

**Learner Audience:**

Medical Students, Interns, Junior Residents, Senior Residents

**Time Required for Implementation:**

This exercise was carried out over two separate conference days. On the first day, it took us two to three hours to complete the SPENT exercise, the small group cases, and large group debrief with the PowerPoint slides; we dedicated a separate conference day for the panel discussion, and we had the opportunity to visit the local shelter and donation center.

**Recommended Number of Learners Per Instructor:**

six to eight learners per instructor

**Topics:**

Social medicine, social determinants of health, homelessness, health literacy, limited English proficiency.

**Objectives:**

The educational objectives of this activity are to:

1. Enhance residents’ understanding and recognition of (SDoH) in emergency medicine.
2. Develop residents’ skills in addressing SDoH within the emergency medicine setting.
3. Foster a patient-centered approach that promotes health equity in emergency medicine.
4. Identify local resources available to address various SDOH.

### Linked objectives and methods

The SDoH curriculum for emergency medicine residents comprises five comprehensive sessions designed to enhance understanding and practical application of SDoH concepts.

Session 1 introduces SDoH through a faculty-led presentation and the SPENT exercise, providing residents with a foundational understanding of how social factors influence health outcomes. Session 2 features case-based simulations where residents work in teams to manage patients with acute medical issues complicated by SDoH factors, encouraging the integration of social considerations into clinical decision-making. Session 3 consists of a faculty-led debriefing, allowing residents to reflect on the challenges and strategies encountered during simulations, fostering deeper insights into the impact of SDoH on patient care. Session 4 involves a panel discussion with social workers and community health workers, equipping residents with knowledge about available community resources and referral networks. Session 5 focuses on interprofessional collaboration, featuring discussions with local shelter managers and a visit to a community donation center. This hands-on experience allows residents to engage directly with community members, utilizing their bilingual skills to bridge language barriers and gain firsthand insights into the challenges faced by individuals affected by SDoH.

### Recommended pre-reading for facilitator

These articles cover a range of topics related to social determinants of health and can provide facilitators with a deeper understanding of the issues involved in conducting a simulation exercise on this topic.

- Raphael D, Mikkonen J. Understanding the social determinants of health: A review of key concepts and principles. *Can J Public Health*. 2010;101(1):9–13.
- Marmot M, Wilkinson RG. The social determinants of health: Coming of age. *Annu Rev Public Health*. 2006;27:399–429.
- Williams DR, Mohammed SA. Racial and ethnic disparities in health: Recent advances and current challenges. *Annu Rev Public Health*. 2013;34:231–258.
- Gottlieb LM, Basu S. Addressing social determinants of health in the clinic setting. *JAMA*. 2016;316(11):1121–1122.

### Learner responsible content (LRC)

Residents were provided reading materials to review prior to the day of the exercise which included a podcast and articles.

- Surendranath S, Rebbeck T. Social Determinants of Health at the Global Level [Internet]. Podcast. Accessed September 7, 2025. Available at: https://sites.libsyn.com/470343/social-determinants-of-health-at-the-global-level Articles:
- Wilkinson R, Marmot M. *Social Determinants of Health: The Solid Facts*. 2nd ed. World Health Organization. Copenhagen: WHO Regional Office for Europe; 2003. NLM Unique ID 101205085.
- Berkowitz SA, Hulberg AC, Hong C, et al. Addressing social determinants of health: time for a polysocial risk score. *J Gen Intern Med*. 2020;35(5):1656–1658.

### Small group application exercise (sGAE)

See the following attached materials for this small group exercise

- Appendix A: Part One: Case-Based Small Group Sessions
  ○ Code Social PowerPoint
  ○ SDoH Case Scenarios
  ○ SDoH Case Scenarios Instructor Guide
- Appendix B: Part Two: Community Resource Panel Discussion

### Results

A total of 16 emergency medicine residents at various training stages achieved a 100% survey completion rate. The survey evaluated residents’ confidence in understanding SDoH, their ability to recognize SDoH impacts on emergency department patients, and their knowledge of related community resources and support services.

*Measure 1: Confidence level of understanding of Social Determinants of Health (SDoH) and their role in healthcare*

- Pre-training average Likert score: 4 out of 5
- Post-training average Likert score: 4.75 out of 5

The Likert score’s increase from 4 to 4.75 suggests an improvement in their confidence level in understanding social determinants of health after the training session.

*Measure 2: Residents’ ability to identify and explain the ways Social Determinants of Health may impact their patients’ physical health, mental well-being, and quality of life*

- Pre-training average Likert score: 3.25 out of 5
- Post-training average Likert score: 4.75 out of 5

After the training session, the average Likert score increased by 1.5 points, which means that there was a significant improvement in the residents’ ability to recognize the various ways SDoH can impact their patient’s well-being.

*Measure 3: Knowledge on local resources for patients of social determinants of health*

- Pre-training average Likert score: 1 out of 5
- Post-training average Likert score: 4.5 out of 5

This measure showed the most substantial improvement, with the average Likert score increasing by 3.5 points. It suggests that the training session effectively equipped residents with strategies for addressing social determinants of health in patient care plans, reflecting a significant enhancement in their skills and competence in this area.

In conclusion, the Simulation Curriculum for Training in SDoH for Emergency Medicine Residents represents a critical step forward in advancing health equity and improving patient care in emergency medicine. Through comprehensive education and practical skill development, residents are better equipped to address the multifaceted challenges posed by social determinants of health and to advocate for systemic changes that promote health and well-being for all individuals and communities.

### Tips for Successful Implementation

Based on our experience, we find it most effective to schedule this session during designated didactics days to ensure maximum attendance from residents. Achieving a 100% survey response rate was facilitated by allowing residents time to complete the survey before leaving the conference room on the same day. Additionally, using a QR code displayed on slides and framing questions in a concise format contributed to this success.

Another key factor in our successful implementation was providing social workers with advance notice of session topics, enabling them to prepare resources and be ready for the panel discussion. We also ensured smooth logistics by pre-arranging visits to the shelter, coordinating with organizers to ensure readiness for our residents’ participation at their distribution center.

To enhance future sessions, we plan to provide the social workers and guest speakers with a predefined set of questions for the panel discussion. This approach aims to foster more diverse and fruitful discussions. We also plan to introduce multiple-choice question (MCQ) testing to measure knowledge acquisition.

## Supplementary Information



## Figures and Tables

**Figure:**
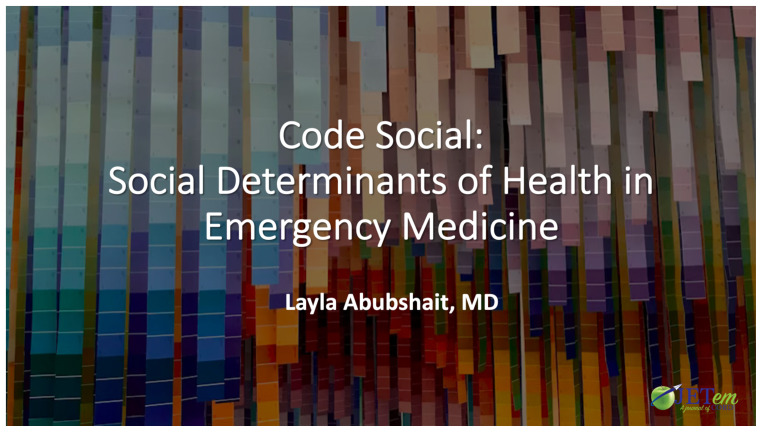
Please see associated PowerPoint file

## SMALL GROUPS LEARNING MATERIALS

### Appendix A Part One: Case-Based Small Group Sessions

We recommend forming small groups based on the total number of learners, with a maximum of eight learners per group to facilitate more engaging discussions. Small groups should remain consistent throughout the session to build group dynamics and trust, while faculty facilitators rotate between cases to ensure all groups receive exposure to different teaching styles and expertise areas.

**Detailed Session Structure:**

**Setup Phase (15 minutes):**

- Divide learners into small groups of six to eight participants each
- Assign one faculty facilitator per group initially
- Distribute case materials and debrief question sheets to each group
- Brief faculty on rotation schedule and timing

**Introduction Phase (30 minutes):**

- Begin with a large group faculty-led introduction to SDoH concepts (15 minutes)
- Conduct the SPENT exercise as a large group activity (15 minutes)
  ○ https://playspent.org/
- This shared foundation ensures all participants start with the same baseline knowledge

**Case Analysis Phase (100 minutes total – 20 minutes per case):**

- Small groups work through each of the five cases systematically
- Faculty facilitators rotate every 20 minutes to a different group
- Each rotation includes:
  ○ Case reading and initial discussion (5 minutes)
  ○ Small group analysis and problem-solving (10 minutes)
  ○ Structured debrief using provided questions (5 minutes)
- Faculty should arrive at each new group with fresh energy and potentially different perspectives on the same case

**Faculty Rotation Benefits:**

- Ensures all groups experience diverse teaching approaches and clinical perspectives
- Prevents faculty fatigue from repeating the same case multiple times
- Allows faculty with specific expertise (eg, social work background, community medicine experience, cultural competency training) to contribute their specialized knowledge across all groups
- Creates consistency in learning outcomes across different groups
- Enables faculty to observe how different groups approach the same clinical scenarios

**Synthesis Phase (15 minutes):**

- Large group discussion where each small group shares one key insight from their case analyses
- Faculty highlight common themes and learning points that emerged across groups
- Address any questions or concerns raised during the small group sessions

**Logistical Considerations:**

- Prepare rotation schedule in advance with clear timing signals (timer, bell, or announcement)
- Brief faculty on smooth transition techniques to minimize disruption
- Consider having one “floating” faculty member to assist with timing and logistics
- Ensure each group has consistent access to materials and workspace throughout rotations

#### SDoH Case Scenarios

**Abbreviations Used in Case Scenarios:**

ED = Emergency Department
EMS = Emergency Medical Services
SDoH = Social Determinants of Health
LEP = Limited English Proficiency
GCS = Glasgow Coma Scale
DM2 = Diabetes Mellitus Type 2
HTN = Hypertension
COPD = Chronic Obstructive Pulmonary Disease
ICU = Intensive Care Unit

**SDoH Case Scenario #1**

**A Case of Limited English Proficiency (LEP)**

Triage Note:

85-year-old female, Bengali speaking, reports chest pain and cough for one month. History of diabetes type 2 (DM2), atrial fibrillation (a-fib). Her son is with her.

ED Course:

You go to speak with the patient, and her son is at bedside translating for her. You offer to get the interpretive services and he declines, stating his mother will prefer if he translates. He answers all your questions, is very familiar with his mother’s past medical history. When you ask review of systems questions, you notice he does not seem to be fully translating your questions to the patient; he is just reporting what the patient has told him the past few days. Based on your history and physical (H&P) you decide to move forward with labs, electrocardiogram (ECG), chest X-Ray (CXR). The CXR is read as having an opacity and associated pleural effusion concerning for a mass/malignant effusion; further characterization by computed tomography (CT) thorax is recommended. You order the CT and discover the patient does indeed have what appears to be a malignancy. You go to inform the patient and her son. The son appears rightfully distraught but says he does not want to tell his mom the results, that she would not want to know and would not want to undergo treatment.

**SDoH Case Scenario #2**

**A Case of Substance Use Disorder and Stigma**

EMS Pre-arrival Report:

21-year-old female found down at a bus stop. Glascow coma scale (GCS) 3, unresponsive, pinpoint pupils, Narcan given twice with minimal effect, bagging on route.

ED Course:

The patient is a 21-year-old female with a known history of depression and polysubstance abuse. Her GCS was 3 initially, improved after Narcan. On arrival she is awake and alert to person, place, and time. She states she is just hungry and asks to leave. She states she doesn’t “care about her life” because, “I don’t have anything to live for anyway, they took my kids away.” She is placed in a room and agrees to be put on end-tidal CO2 if she can get some food. After four hours of observation, the patient falls asleep. Her end-tidal CO_2_ remains normal. When awakened, she requests intravenous pain medication, stating her feet hurt. You notice her feet are never examined by the team. She mentions concerns about possible cellulitis but is told the wounds are just scrapes from her fall. She complains about not receiving food despite watching dinner trays being distributed to other patients. The medical record documents “patient exhibits drug-seeking behavior.”

During disposition planning, the nurse tells the attending physician, “I know her. She’s always here and never listens. She’s a well-known addict. Don’t even bother giving her a sandwich— she’ll just ask for pain medicine and get aggressive when she doesn’t get it.” The attending agrees, noting the patient is well known to ED staff, and prepares discharge paperwork. The resident prescribes naloxone, which is sent to the patient’s pharmacy, and advises her to stop using drugs or she might die. The patient requests food, explaining she has a long trip ahead and is currently homeless. She is given crackers and ginger ale. It is late at night, so she is offered a ride home. She refuses, stating she never received adequate food and never spoke with the attending physician. She asks about buprenorphine therapy, explaining this is her seventh overdose and she fears she will not recover. She is told she must be in active withdrawal to initiate treatment. She becomes angry, stating staff do not understand what she is going through.

The urine drug screen remains pending, and psychiatry is not consulted. Other laboratory results are normal and vital signs are stable, though she has a mildly elevated temperature that is not documented. Her discharge is prioritized due to a crowded waiting room. After waiting an additional two hours, she is escorted out by security when she refuses to leave the hospital bed following discharge.

One week later, the patient overdoses again and experiences cardiac arrest in the ambulance. She is pronounced dead on arrival at the ED, still wearing the same yellow socks from her previous visit.

**SDoH Case Scenario #3**

**A Case of Health Literacy**

Triage Note:

A 68-year-old male with cough, fever, and respiratory distress.

ED Course:

A 68-year-old male, a smoker, arrived at the ED complaining of a week-long cough and shortness of breath, now accompanied by blood-tinged sputum. On examination, he presented with fever, tachycardia, and oxygen saturation of 87% on room air, with bilateral crackles noted on lung auscultation. A chest x-ray revealed multifocal pneumonia, and his lab results indicated leukocytosis with a count of 22,000.

During history-taking, the patient denied any medical issues, though his problem list indicated hypertension (HTN) and chronic obstructive pulmonary disease (COPD), with his last medication fill noted in 2019 following an ED visit. Reviewing his chart revealed a history of being labeled a “poor historian” and “non-compliant,” with no designated family doctor. You informed the patient that he has pneumonia and due to his oxygen levels, you recommended that he be admitted to the hospital; patient laughs and tells you he feels fine and better after the “oxygen therapy” and wants to go home.

**SDoH Case Scenario #4**

**A Case of Substance Use Disorder and Bias**

Pre-arrival Report:

Emergency medical services (EMS) responded to a call for a 35-year-old female who experienced seizure-like activity and is now in a post-ictal state.

ED Course:

The patient arrives accompanied by her 12-year-old daughter. She is immediately placed on cardiac monitoring with vital signs within normal limits. Although alert and oriented to person, place, and time, she appears anxious. She responds appropriately to questions and reports headache as her only complaint.

According to her daughter, she was in another room when she heard a loud noise. Upon investigation, she found her mother having a tonic-clonic seizure with foaming at the mouth. The patient reports a history of seizures and states she is compliant with her medications. She denies alcohol use, admits to smoking cigarettes, and denies illicit drug use.

You observe a change in the patient’s demeanor and body language when questioned about drug use. You decide to escort her privately to the restroom for further discussion, away from her daughter. During this private conversation, she tearfully admits to snorting cocaine and fentanyl prior to the seizure. She requests this information remain confidential from her daughter.

While documenting at the workstation, you overhear a group of ED nurses making disparaging comments about the patient. They express sentiments blaming her for the overdose and question her fitness for custody of her children. You feel uncomfortable with these comments but do not feel empowered to address the situation directly.

**SDoH Case Scenario #5**

**A Case of Cultural Beliefs and LEP**

ED Course:

A 62-year-old Spanish-speaking man with a history of type 2 diabetes mellitus controlled with oral medications presented to the emergency department complaining of a headache and blurry vision. Evaluation revealed a hypertensive emergency with a notable increase in creatinine levels.

You recommend arterial line placement and ICU admission for careful blood pressure management. The patient expresses reluctance to be admitted. Despite your explanation of potential risks, including the possibility of requiring dialysis if he leaves against medical advice, the patient remains steadfast in his decision. He expresses his faith in God and states he is ready to accept whatever outcome awaits him.

As you leave the patient’s room, you comment to the medical student, “I don’t understand people like that—but if he wants God to save him, that’s fine. I won’t waste my time trying.”

#### SDoH Case Scenarios Instructor Guide

**Abbreviations Used in Case Scenarios:**

ED = Emergency Department
EMS = Emergency Medical Services
SDoH = Social Determinants of Health
LEP = Limited English Proficiency
GCS = Glasgow Coma Scale
DM2 = Diabetes Mellitus Type 2
HTN = Hypertension
COPD = Chronic Obstructive Pulmonary Disease
ICU = Intensive Care Unit

**Case Scenario #1: Limited English Proficiency (LEP)**

**Clinical Scenario Summary:** An 85-year-old Bengali-speaking female presents with chest pain and cough. Her son serves as interpreter and later refuses to inform her of malignant findings on imaging.

**Debrief Questions and Main Teaching Points:**

1. How did the language barrier affect the initial assessment and communication with the patient?
  *Main teaching points: Language barriers lead to incomplete or inaccurate medical histories, missed symptoms, and inadequate informed consent. Family translators may filter information, omit sensitive topics, or lack medical terminology knowledge. This compromises patient autonomy and quality of care.*
2. What are the potential consequences of using family members as translators in healthcare settings?
  *Main teaching points: Family translators may have conflicts of interest, lack objectivity, may not convey sensitive information accurately, and can compromise patient autonomy. Professional interpreters are trained to remain neutral, maintain confidentiality, and provide accurate translation of medical terminology.*
3. How might the patient’s experience and outcomes differ if professional interpreter services were utilized?
  *Main teaching points: Professional interpreters ensure accurate bidirectional communication, maintain patient confidentiality, support true informed consent, and preserve the therapeutic relationship. They enable patients to participate fully in their care decisions and understand their diagnoses and treatment options.*
4. What are the legal and ethical obligations for providing language access services to patients with LEP?
  *Main teaching points: Hospitals are legally required to provide interpreter services under Title VI of the Civil Rights Act. Healthcare providers must know how to access these services, understand cultural competency principles, and recognize that language access is fundamental to health equity and quality care.*

**Case Scenario #2: Substance Use Disorder and Stigma**

**Clinical Scenario Summary:** A 21-year-old female with polysubstance use disorder presents after naloxone-reversed overdose. Despite multiple unmet needs and requests for addiction treatment, she receives stigmatizing care and is discharged. She dies from overdose one week later.

**Debrief Questions and Main Teaching Points:**

1. How might the patient’s basic needs for food and pain assessment have been better addressed during her ED visit?
  *Main teaching points: Basic human needs like nutrition should be addressed regardless of patient demographics or history. Pain requires objective assessment using validated tools. Homelessness and substance use disorder are medical conditions deserving compassionate, evidence-based care.*
2. Discuss the impact of labeling the patient as a “well-known addict” and dismissing her concerns as drug-seeking behavior. How does this affect care quality and patient outcomes?
  *Main teaching points: Stigmatizing labels create implicit bias affecting clinical decision-making. They lead to substandard care, missed diagnoses, and erosion of therapeutic relationships. Healthcare providers should use person-first language and provide evidence-based care regardless of substance use history.*
3. Given the patient’s seven previous overdoses and explicit request for buprenorphine therapy, how could the healthcare team have better supported her recovery?
  *Main teaching points: Substance use disorder is a chronic, treatable medical condition requiring intervention, not moral judgment. Emergency departments can initiate medication-assisted treatment, provide harm reduction resources, and facilitate warm handoffs to outpatient services. ED-initiated buprenorphine significantly improves treatment engagement and reduces mortality.*
4. What system-level changes could improve care for patients with substance use disorder and co-occurring mental health conditions?
  *Main teaching points: Implement trauma-informed care principles, provide staff education on implicit bias and stigma reduction, establish protocols for substance use disorder screening and treatment initiation, ensure access to psychiatric consultation and social services, and create pathways for addiction treatment referrals.*

**Case Scenario #3: Health Literacy**

**Clinical Scenario Summary:** A 68-year-old male smoker with HTN and COPD presents with severe pneumonia requiring admission. He misunderstands his treatment and insists on discharge after receiving “oxygen therapy.”

**Debrief Questions and Main Teaching Points:**

1. How did the patient’s health literacy level affect his understanding of his diagnosis and treatment needs?
  *Main teaching points: Low health literacy leads to misunderstanding of medical conditions, treatment plans, and disease severity. Patients may not comprehend the consequences of refusing recommended care. The “oxygen therapy” comment demonstrates misunderstanding of both his disease process and treatment requirements.*
2. What communication strategies could ensure the patient understands the severity of his condition and the necessity of hospital admission?
  *Main teaching points: Use plain language avoiding medical jargon, employ teach-back methods (“Can you explain back to me what we discussed?”), utilize visual aids when available, assess understanding frequently, and explain risks of leaving against medical advice in concrete, understandable terms.*
3. Discuss the importance of informed consent and shared decision-making when a patient wishes to leave against medical advice.
  *Main teaching points: Patients have autonomy to make informed decisions about their care, but true informed consent requires comprehension. Healthcare providers must balance respect for autonomy with ensuring patients understand the consequences of their decisions. Document capacity assessment and attempt to address barriers to acceptance of care.*
4. What community resources and interventions could improve this patient’s health literacy and address barriers to medication compliance?
  *Main teaching points: Community health workers, patient navigators, health literacy programs, and accessible follow-up care bridge gaps in understanding. Address barriers including lack of insurance, transportation limitations, medication costs, and absence of primary care relationships. Simplified discharge instructions and follow-up phone calls improve outcomes.*
5. How can healthcare providers adapt their communication and education approaches for patients with varying health literacy levels?
  *Main teaching points: Assess health literacy using validated screening tools or clinical observation, tailor communication to individual needs, use multiple teaching modalities, provide written materials at appropriate reading levels (typically 5th–6th grade), incorporate teach-back universally, and arrange close follow-up to reinforce understanding.*

**Case Scenario #4: Substance Use Disorder and Bias**

**Clinical Scenario Summary:** A 35-year-old woman presents post-seizure with her daughter present. She privately discloses cocaine and fentanyl use while staff make judgmental comments about her parenting fitness within earshot.

**Debrief Questions and Main Teaching Points:**

1. How did the patient’s concern about disclosure in front of her daughter impact the clinical encounter and information gathering?
  *Main teaching points: Substance use stigma prevents honest disclosure, particularly when children are present. Healthcare providers must create safe spaces for sensitive conversations while being mindful of family dynamics. Private discussions may be necessary to obtain accurate histories and provide appropriate counseling.*
2. Analyze the ED nurses’ disparaging comments about the patient. What are the implications for patient trust, future healthcare-seeking behavior, and team professionalism?
  *Main teaching points: Judgmental attitudes from any healthcare team member damage therapeutic relationships, discourage help-seeking behavior, and perpetuate health disparities. All team members require education about implicit bias, professional communication standards, and the medical nature of addiction. Such comments may be overheard and cause psychological harm.*
3. What strategies can healthcare teams implement to create non-judgmental environments for patients disclosing substance use?
  *Main teaching points: Implement trauma-informed care principles, provide regular staff training on bias reduction and addiction as disease, establish clear policies about professional conduct, use motivational interviewing techniques, and create a culture where staff feel empowered to address unprofessional behavior when witnessed.*
4. How should healthcare providers balance patient privacy rights with concerns about child welfare?
  *Main teaching points: Adult patients have privacy rights protected by HIPAA, even from family members. Healthcare providers should offer private time for sensitive discussions.*
  *Mandatory reporting requirements apply only when there is reasonable suspicion of child abuse or neglect. Substance use alone does not automatically constitute neglect requiring reporting.*
5. Discuss how SDoH including stigma, social support networks, and healthcare provider attitudes affect patient outcomes in substance use disorder.
  *Main teaching points: Stigma is a powerful social determinant negatively affecting health outcomes through multiple pathways: reducing treatment-seeking, damaging self-efficacy, limiting social support, and causing discrimination in healthcare settings. Building trust, providing nonjudgmental care, addressing implicit bias, and connecting patients with appropriate resources are essential for improving outcomes.*

**Case Scenario #5: Cultural Beliefs and Limited English Proficiency**

**Clinical Scenario Summary:** A 62-year-old Spanish-speaking man with DM2 presents with hypertensive emergency and acute kidney injury. He declines ICU admission based on religious faith. The resident makes dismissive comments about the patient’s beliefs.

**Debrief Questions and Main Teaching Points:**

1. How did the patient’s cultural beliefs and faith influence his decision to decline recommended hospital admission?
  *Main teaching points: Cultural and religious beliefs significantly shape health decisions and interpretations of illness. Faith-based perspectives on healing and acceptance of outcomes are valid and deserve respect. Healthcare providers require cultural competency to work effectively with diverse populations and belief systems.*
2. What barriers might this patient face in understanding and navigating the healthcare system given his language preference and cultural background?
  *Main teaching points: Language barriers, cultural differences in healthcare expectations and communication styles, varying health belief models, different conceptualizations of illness causation, and potential historical mistrust of medical systems all impact care. These barriers contribute to delayed care, poor outcomes, and health disparities.*
3. What communication strategies ensure informed decision-making and mutual understanding with culturally diverse patients?
  *Main teaching points: Use professional interpreters for all clinical encounters, learn about common cultural health beliefs in your patient population, demonstrate respect for diverse perspectives, involve cultural liaisons or community health workers when available, and invest time in understanding the patient’s worldview and values. Professional interpreters often provide cultural context beyond language translation.*
4. How can healthcare teams respectfully integrate cultural beliefs into care plans while ensuring patient safety?
  *Main teaching points: Practice collaborative care planning incorporating cultural preferences, involve family and spiritual leaders when patient desires, seek common ground between medical recommendations and cultural beliefs, provide culturally sensitive education about medical conditions and treatments, and explore alternative approaches that respect beliefs while optimizing health outcomes. Distinguish between respecting autonomy versus abandoning patients.*
5. Reflect on the resident’s dismissive comment. How do provider attitudes about cultural and religious beliefs affect patient care and the learning environment?
  *Main teaching points: Dismissive attitudes toward patient beliefs damage therapeutic relationships, model unprofessional behavior to trainees, and reflect cultural incompetence. Healthcare providers must examine their own biases and develop cultural humility. Such comments are inappropriate and may constitute unprofessional conduct requiring intervention from supervisors.*

### Appendix B Part Two: Community Resource Panel Discussion

**Overview:** Part Two should be conducted on a different conference day from the case-based sessions, ideally within 1–2 weeks of Part One. This separation allows residents to process and reflect on the case scenarios before engaging with community resources and provides adequate time to coordinate with multiple community stakeholders. Part Two involves a structured panel discussion with community stakeholders including social workers, local shelter managers, community health workers, and other relevant service providers. This session bridges the gap between theoretical case discussions and real-world resource availability.

**Panel Composition: based on what is available to your program**

- Hospital-based social workers (one to two representatives)
- Local homeless shelter managers or coordinators (one to two representatives)
- Community health center representatives
- Representatives from local food assistance programs
- Mental health and substance abuse treatment facility coordinators
- Legal aid or patient advocacy representatives
- Transportation assistance program coordinators

**Pre-Panel Preparation:**

- Contact panelists two-three weeks in advance to explain session objectives
- Provide panelists with case scenarios in advance for context
- Share predefined discussion questions to allow preparation
- Request that panelists bring informational materials, brochures, and contact information
- Coordinate logistics including seating arrangement, microphones, and presentation capabilities

**Identifying and Recruiting Panel Members:** Hospital-based social workers and case managers serve as invaluable resources for connecting with community partners. They maintain established relationships with local shelter managers, community organization leaders, and service providers through their daily discharge planning and patient advocacy work. We recommend starting the recruitment process by consulting with your hospital’s social work department to identify their most frequently used community partners, and leveraging social workers’ knowledge of those community leaders who are most effective communicators and comfortable in educational settings.

**Session Structure (60 minutes total):**

**Introduction Phase (10 minutes):**

- Faculty facilitator introduces panel members and their organizational roles
- Brief overview of session objectives and format
- Ground rules for respectful dialogue and Q&A protocol

**Structured Panel Discussion (35 minutes):** Using predefined questions that relate directly to the case scenarios:

1. **Resource Identification (10 minutes):**
  ○ “Based on the cases discussed today, what specific resources does your organization provide that could have helped these patients?”
  ○ “What are the eligibility requirements and application processes for your services?”
2. **Referral Processes (10 minutes):**
  ○ “How can emergency department staff connect patients with your services?”
  ○ “What information do you need from healthcare providers to facilitate referrals?”
  ○ “Are there 24/7 contact methods for urgent situations?”
3. **Common Barriers and Solutions (10 minutes):**
  ○ “What are the most common barriers patients face when trying to access your services?”
  ○ “How can healthcare providers help patients overcome these barriers?”
  ○ “What misconceptions do healthcare providers often have about your services?”
4. **Collaboration Opportunities (5 minutes):**
  ○ “How can we improve communication between the emergency department and community organizations?”
  ○ “What would an ideal referral relationship look like?”

**Interactive Q&A Session (15 minutes):**

- Residents ask specific questions based on their case discussions
- Panel members provide detailed responses and practical guidance
- Focus on actionable steps residents can take in clinical practice

**Resource Distribution and Contact Exchange:**

- Panelists distribute printed materials and business cards
- Create a shared contact list for all participants
- Provide residents with quick-reference cards for common resources

**Implementation Tips for Panel Success:**

**Advance Planning:**

- Schedule panel during protected didactic time to ensure resident attendance
- Confirm panelist availability and backup participants
- Set up room to facilitate interaction (U-shape or round table preferred)

**Optional Community Site Visit:** Our program was able to arrange for residents to visit a local shelter and donation center, which significantly enhanced the residents’ experience by providing firsthand exposure to community resources and the populations they serve. While this site visit is not necessary for achieving the learning objectives, it proved to be a valuable activity that deepened residents’ understanding of resource availability and barriers patients face. If logistically feasible, consider coordinating with panel participants to arrange visits to their facilities either before or after the panel discussion. This hands-on experience allows residents to see resources in action and better understand referral processes.

**Follow-up Activities:**

- Distribute digital contact list within 48 hours
- Create quick-reference resource guide based on panel information
- Schedule periodic updates as community resources change
- Consider establishing ongoing communication channels between ED and community partners
