# Supplementary material for: Code Social: Integrating SDoH into Emergency Resident Education
Source: J Educ Teach Emerg Med. 2025 Oct 31;10(4):SG87–SG113. doi: 10.21980/J8.52163 (PMC12594463; doi:10.21980/J8.52163)
Supplement: Supplementary file 1 [file 10-4-SG87-Supp1.pptx]

## Slide 1
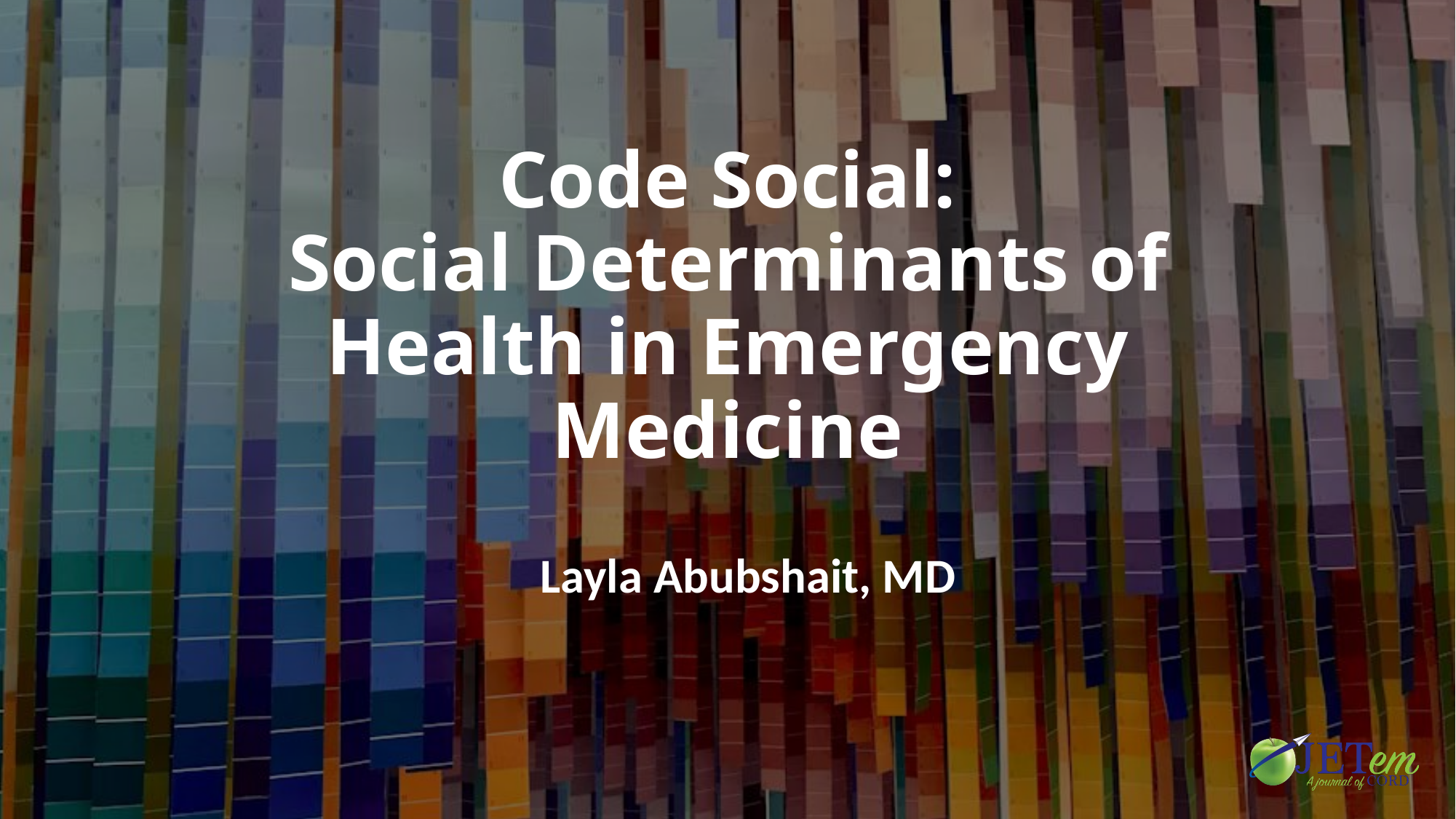

# Code Social: Social Determinants of Health in Emergency Medicine
Layla Abubshait, MD

## Slide 2
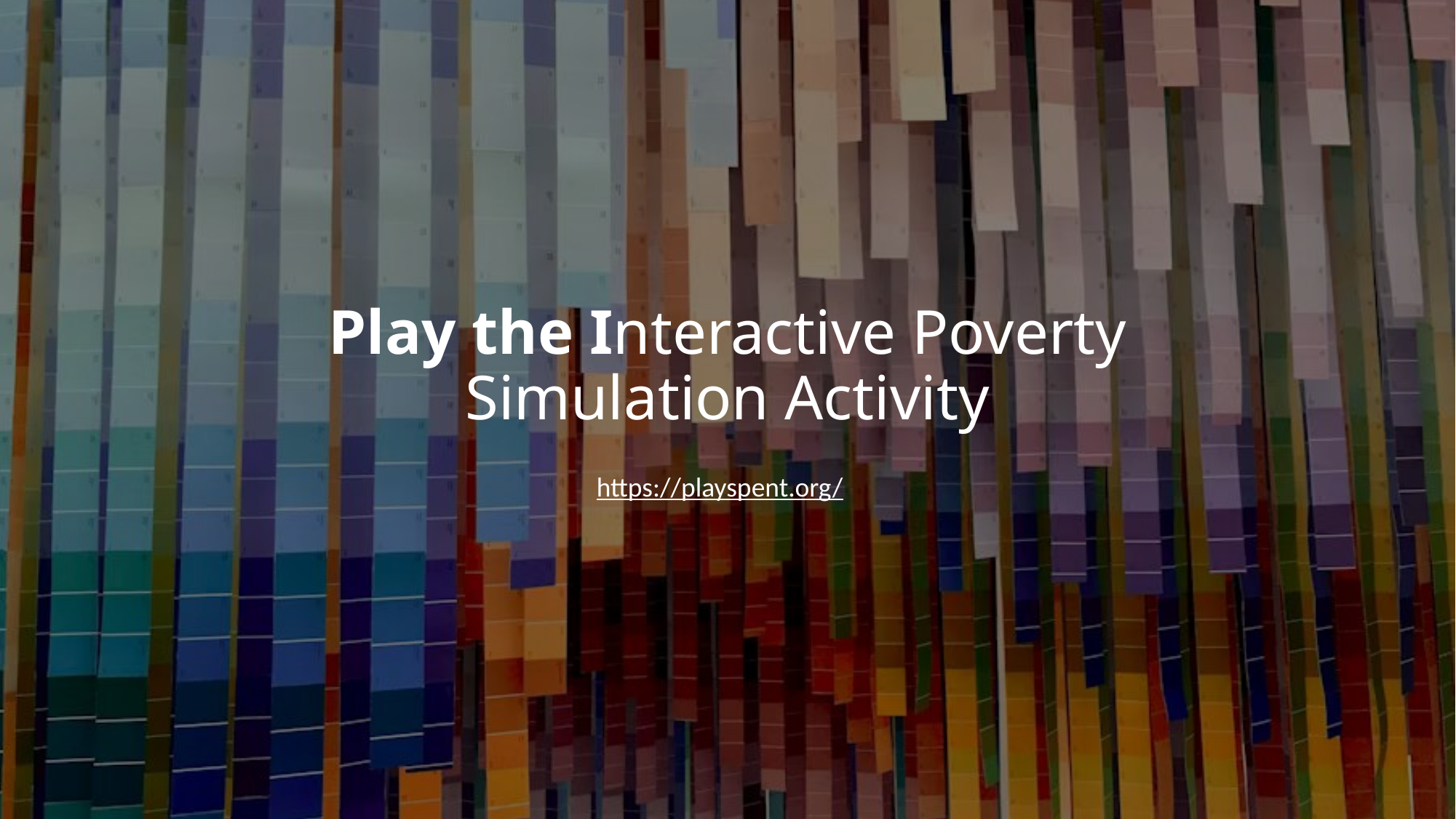

# Play the Interactive Poverty Simulation Activity
https://playspent.org/
2

## Slide 3
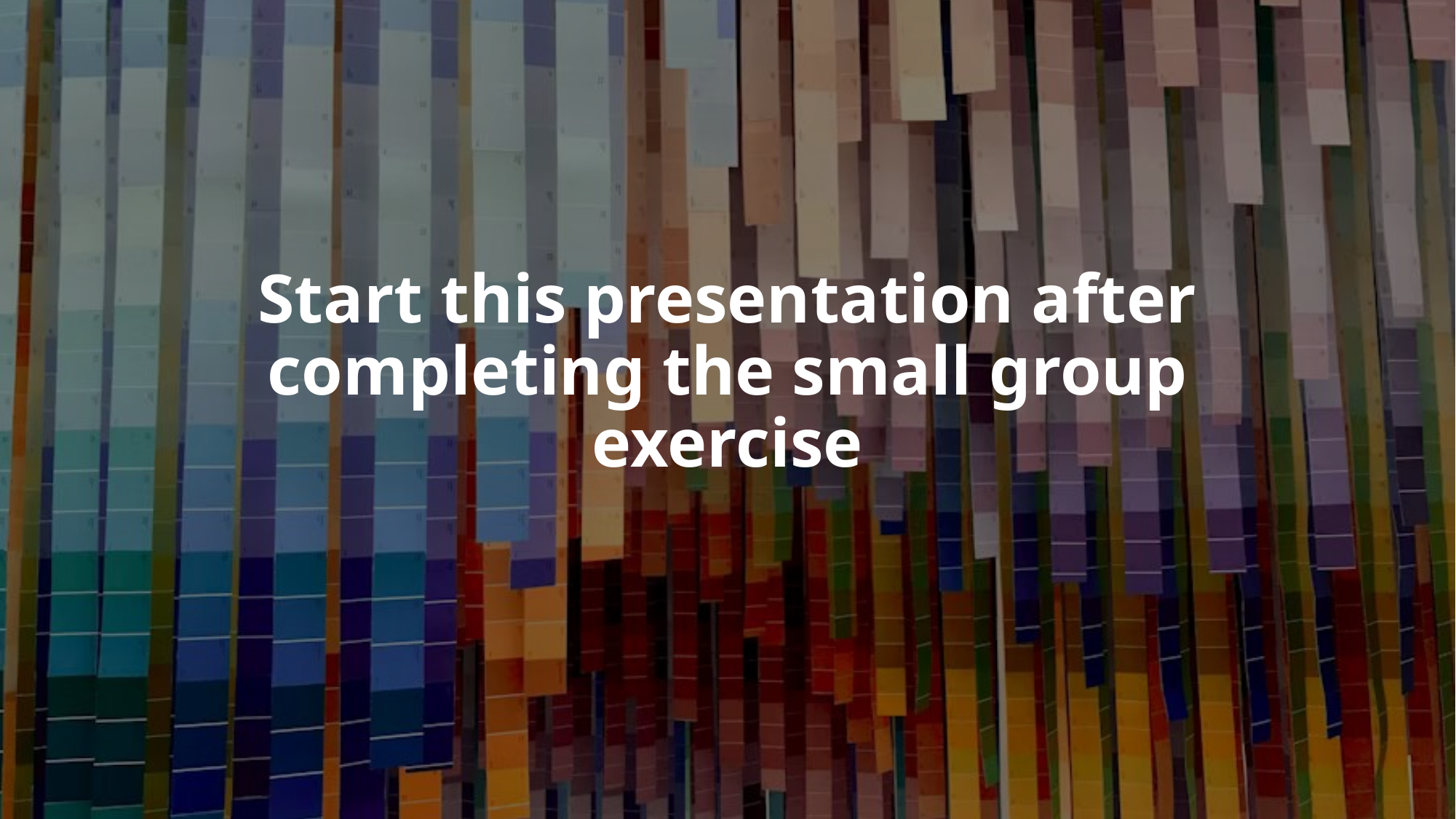

# Start this presentation after completing the small group exercise
3

## Slide 4
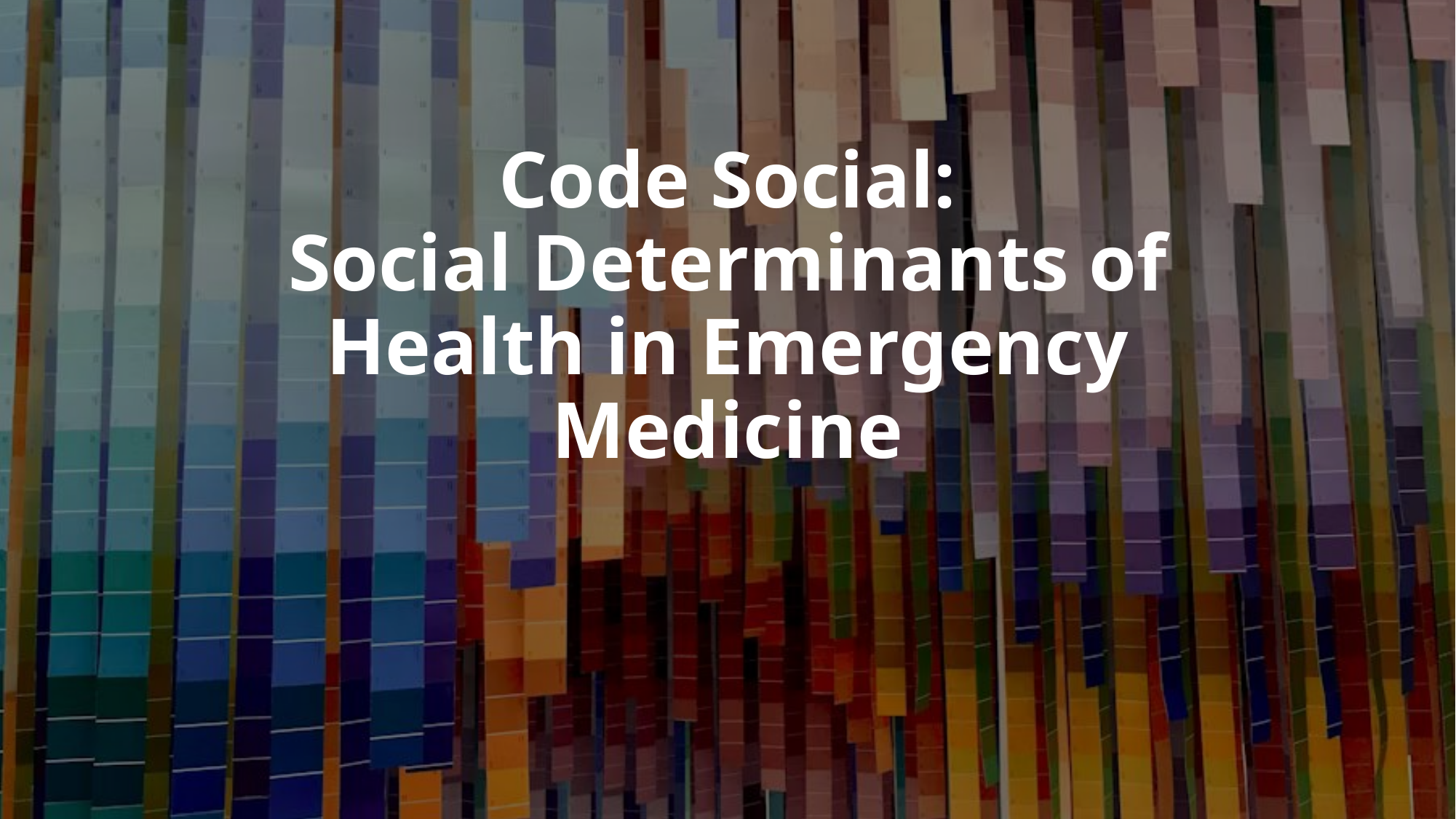

# Code Social: Social Determinants of Health in Emergency Medicine
4

## Slide 5
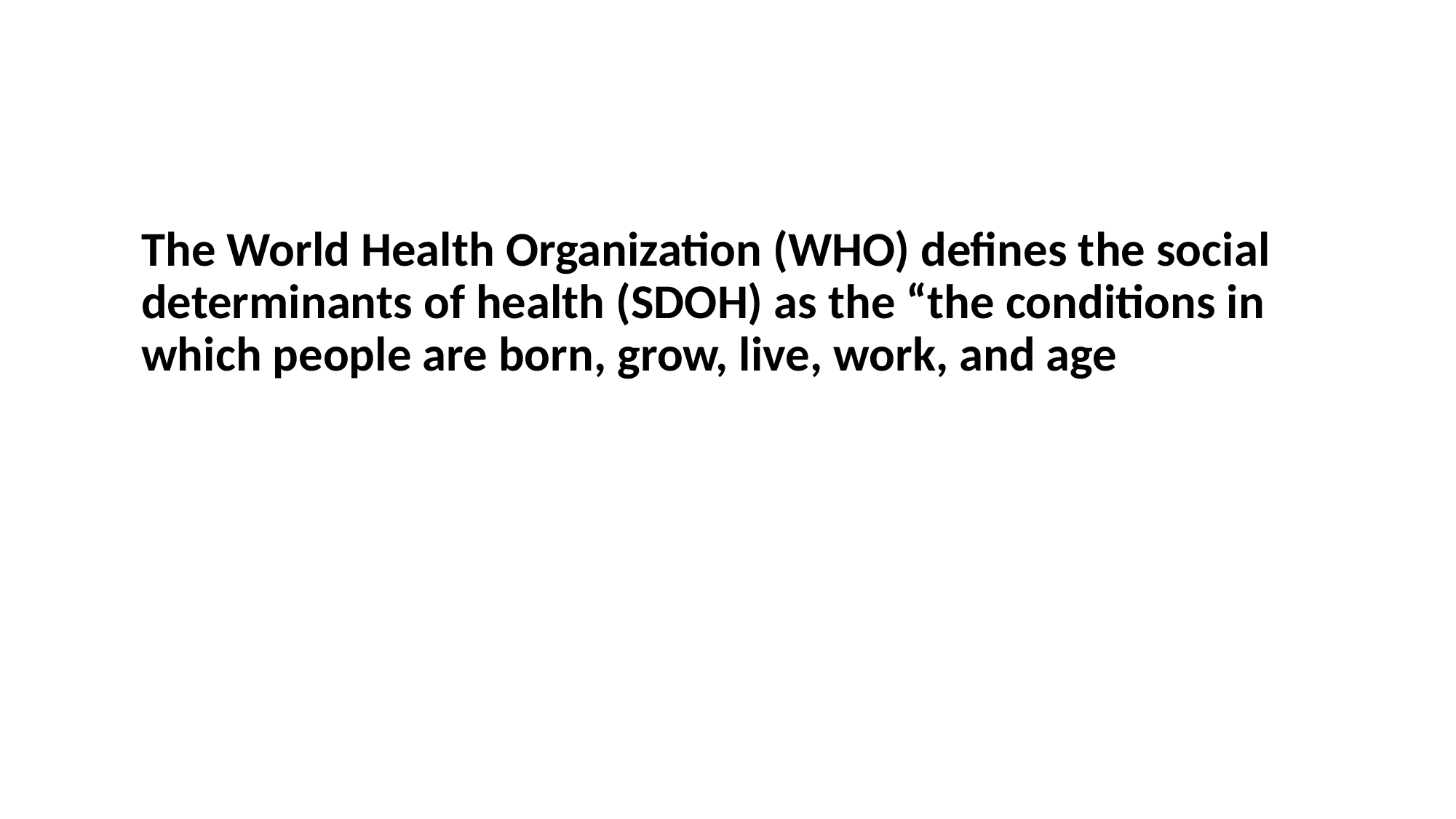

The World Health Organization (WHO) defines the social determinants of health (SDOH) as the “the conditions in which people are born, grow, live, work, and age
5

## Slide 6
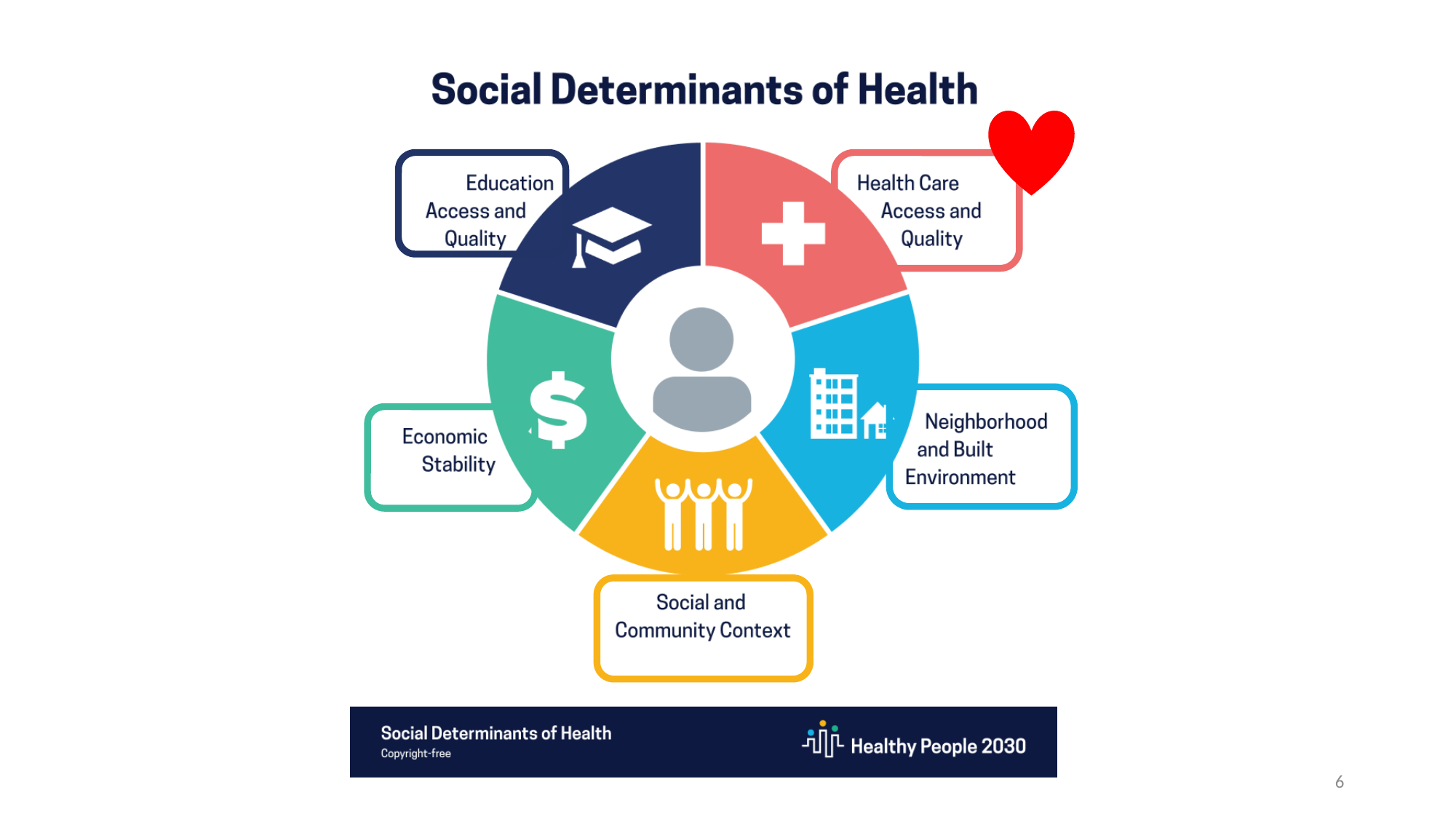

6

## Slide 7
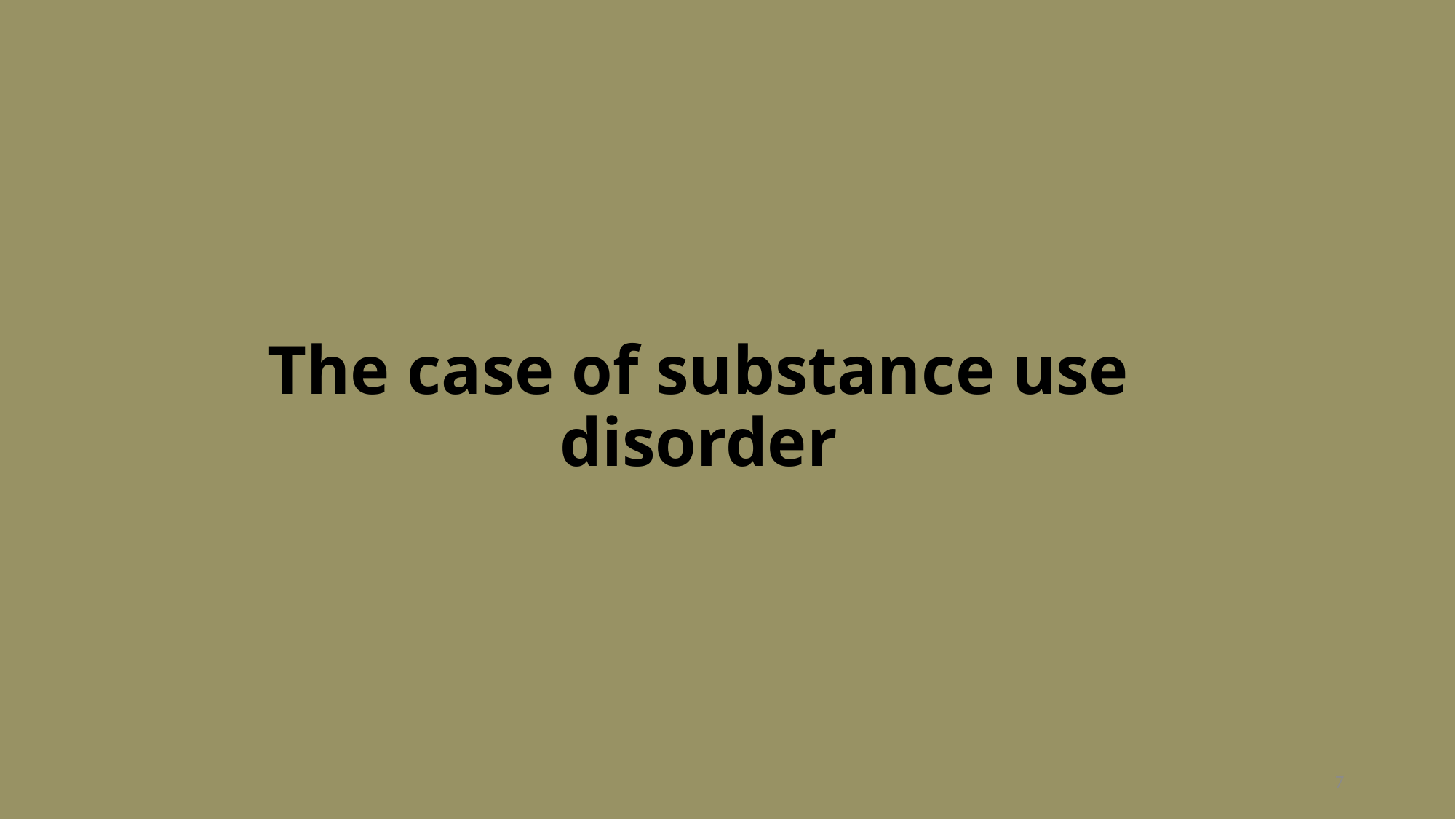

# The case of substance use disorder
7

## Slide 8
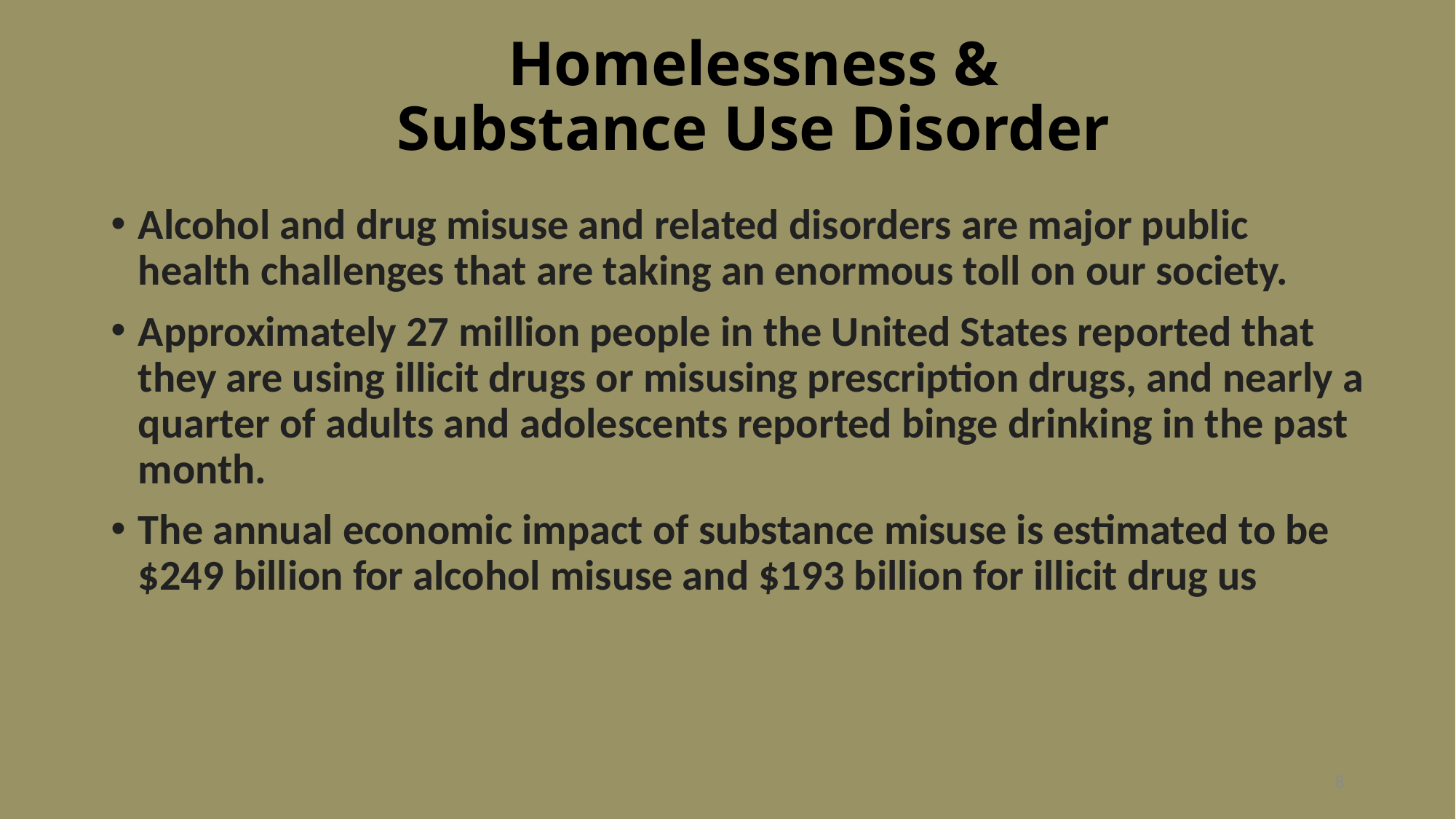

# Homelessness & Substance Use Disorder
Alcohol and drug misuse and related disorders are major public health challenges that are taking an enormous toll on our society.
Approximately 27 million people in the United States reported that they are using illicit drugs or misusing prescription drugs, and nearly a quarter of adults and adolescents reported binge drinking in the past month.
The annual economic impact of substance misuse is estimated to be $249 billion for alcohol misuse and $193 billion for illicit drug us
8

## Slide 9
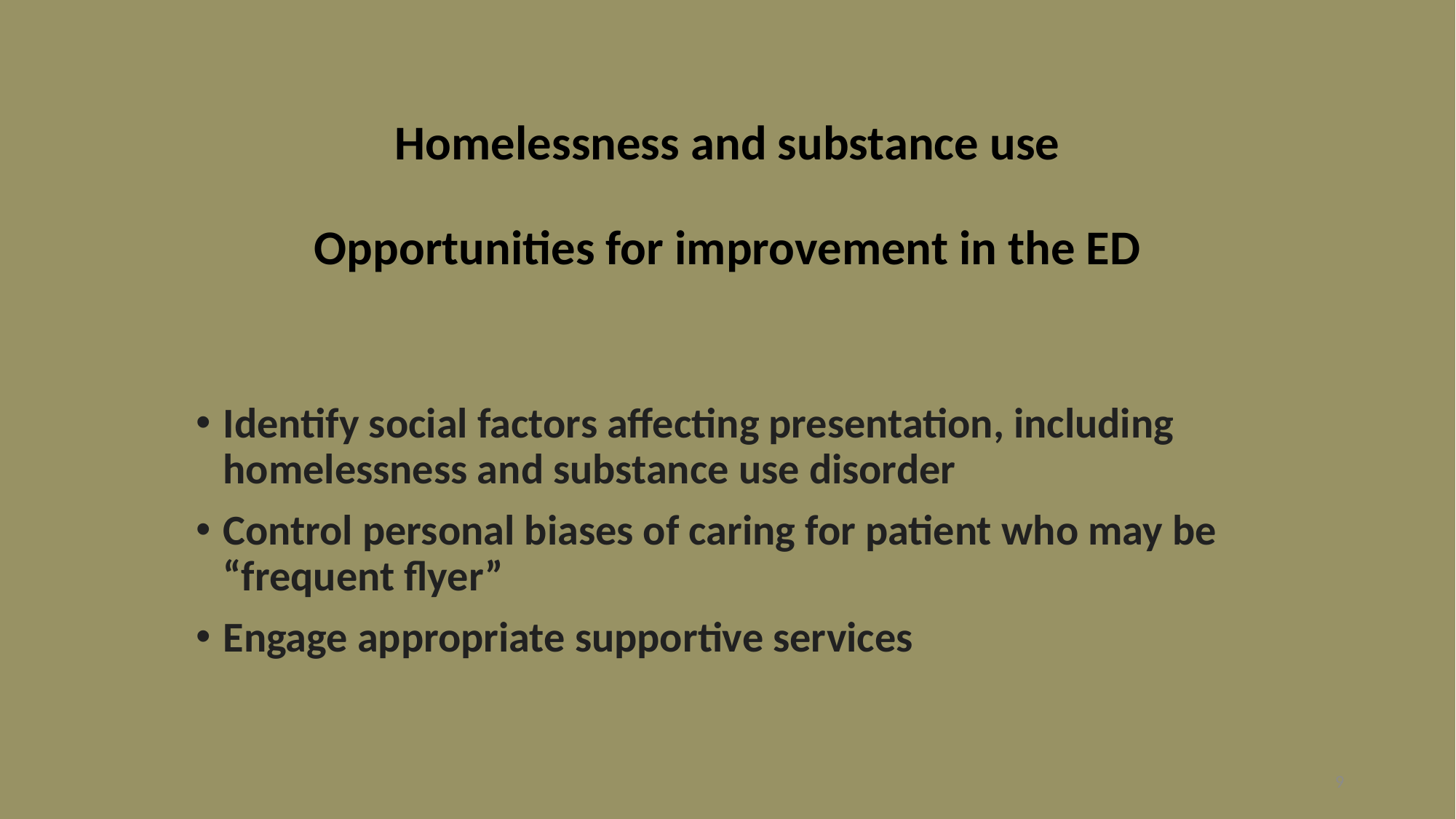

# Homelessness and substance use Opportunities for improvement in the ED
Identify social factors affecting presentation, including homelessness and substance use disorder
Control personal biases of caring for patient who may be “frequent flyer”
Engage appropriate supportive services
9

## Slide 10
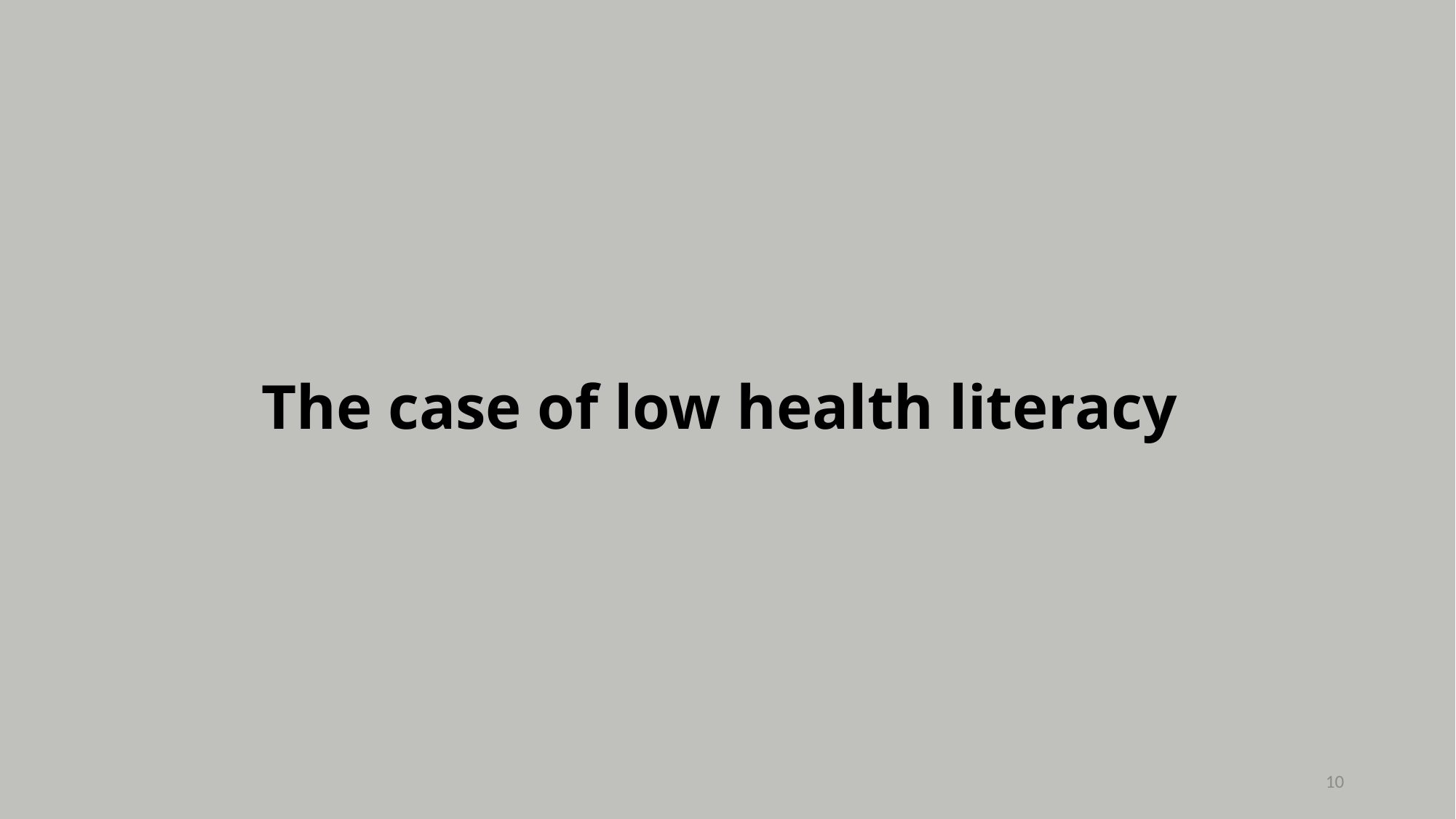

# The case of low health literacy
10

## Slide 11
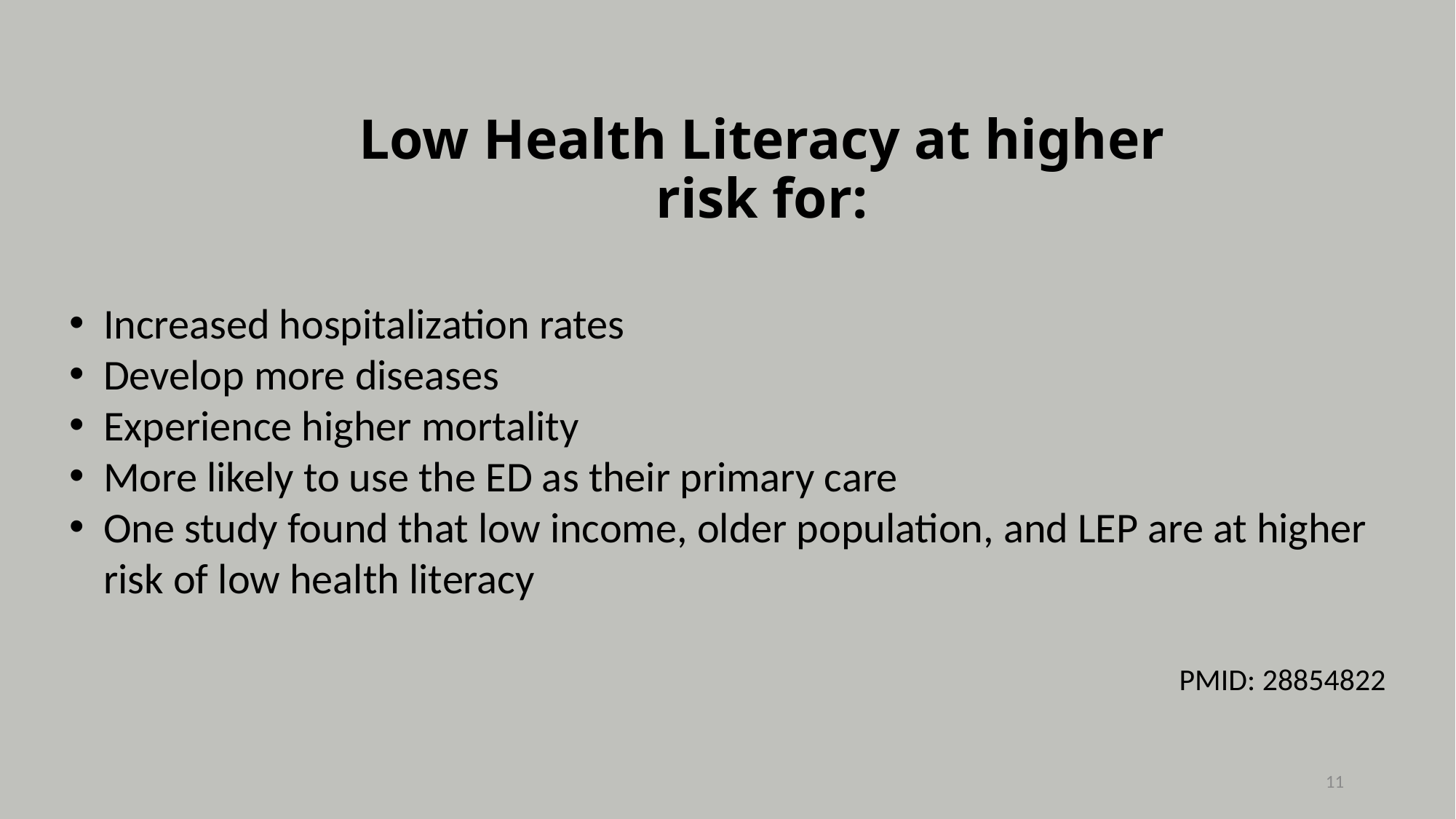

# Low Health Literacy at higher risk for:
Increased hospitalization rates
Develop more diseases
Experience higher mortality
More likely to use the ED as their primary care
One study found that low income, older population, and LEP are at higher risk of low health literacy
PMID: 28854822
11

## Slide 12
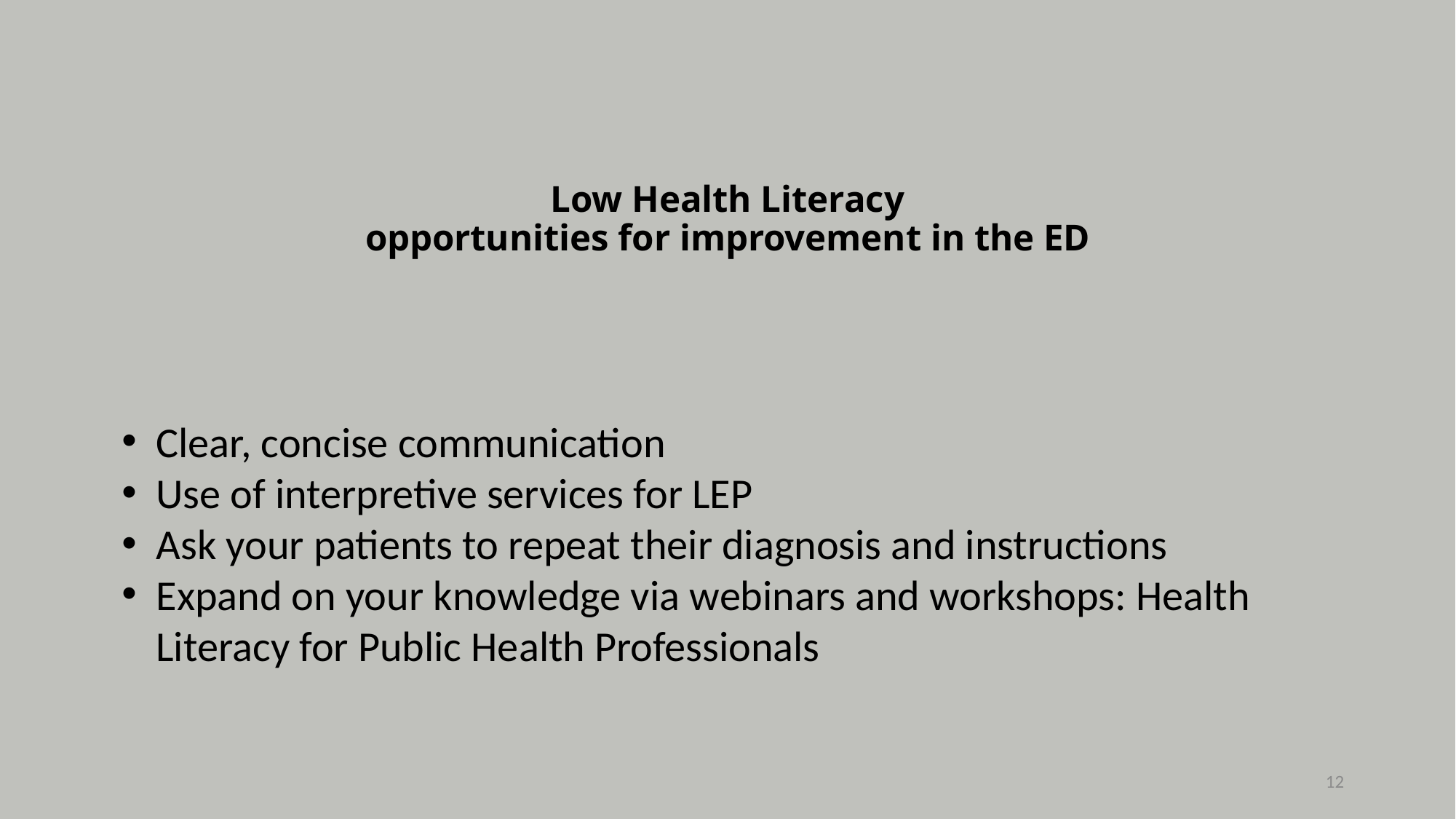

# Low Health Literacy opportunities for improvement in the ED
Clear, concise communication
Use of interpretive services for LEP
Ask your patients to repeat their diagnosis and instructions
Expand on your knowledge via webinars and workshops: Health Literacy for Public Health Professionals
12

## Slide 13
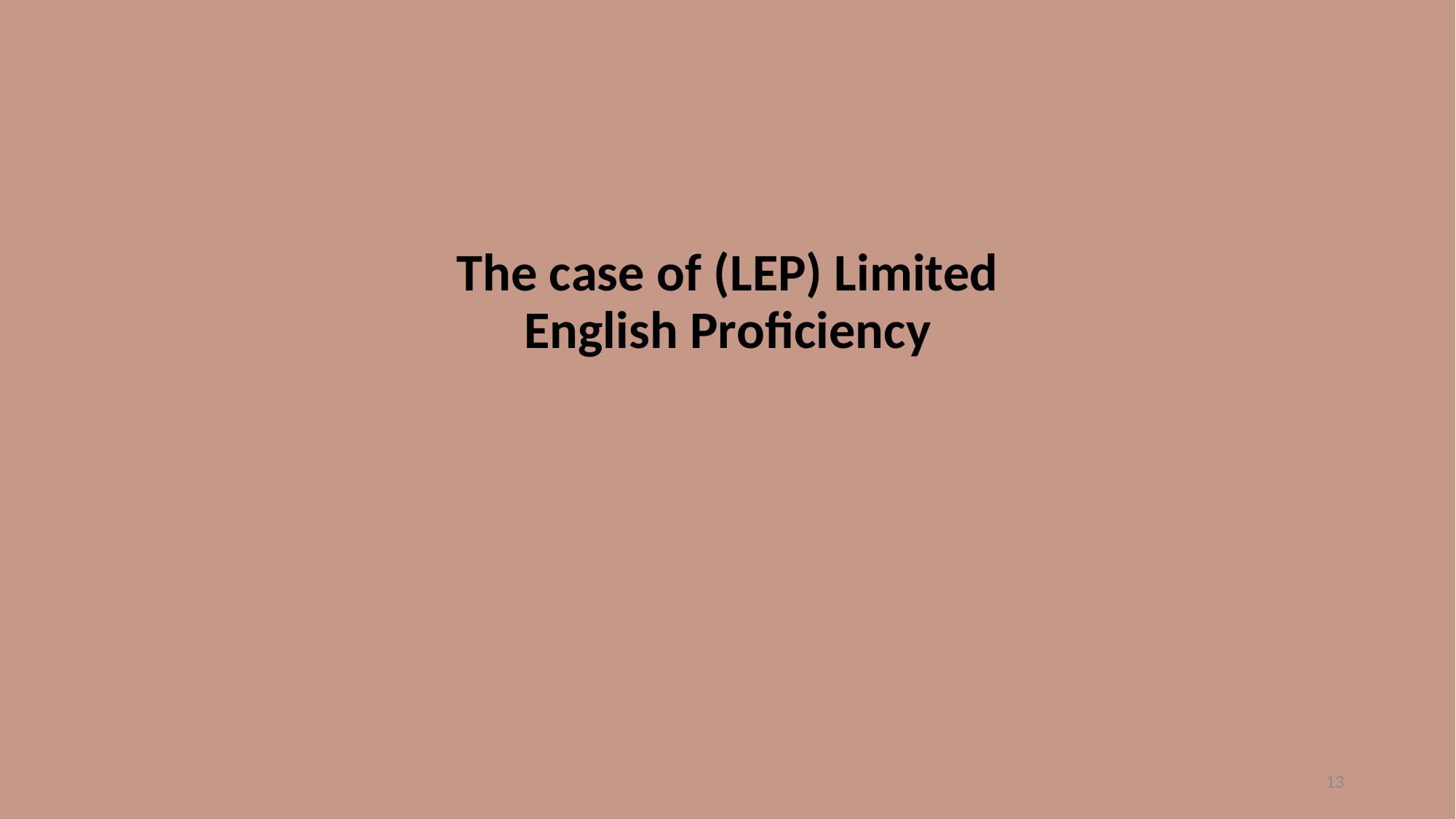

# The case of (LEP) Limited English Proficiency
13

## Slide 14
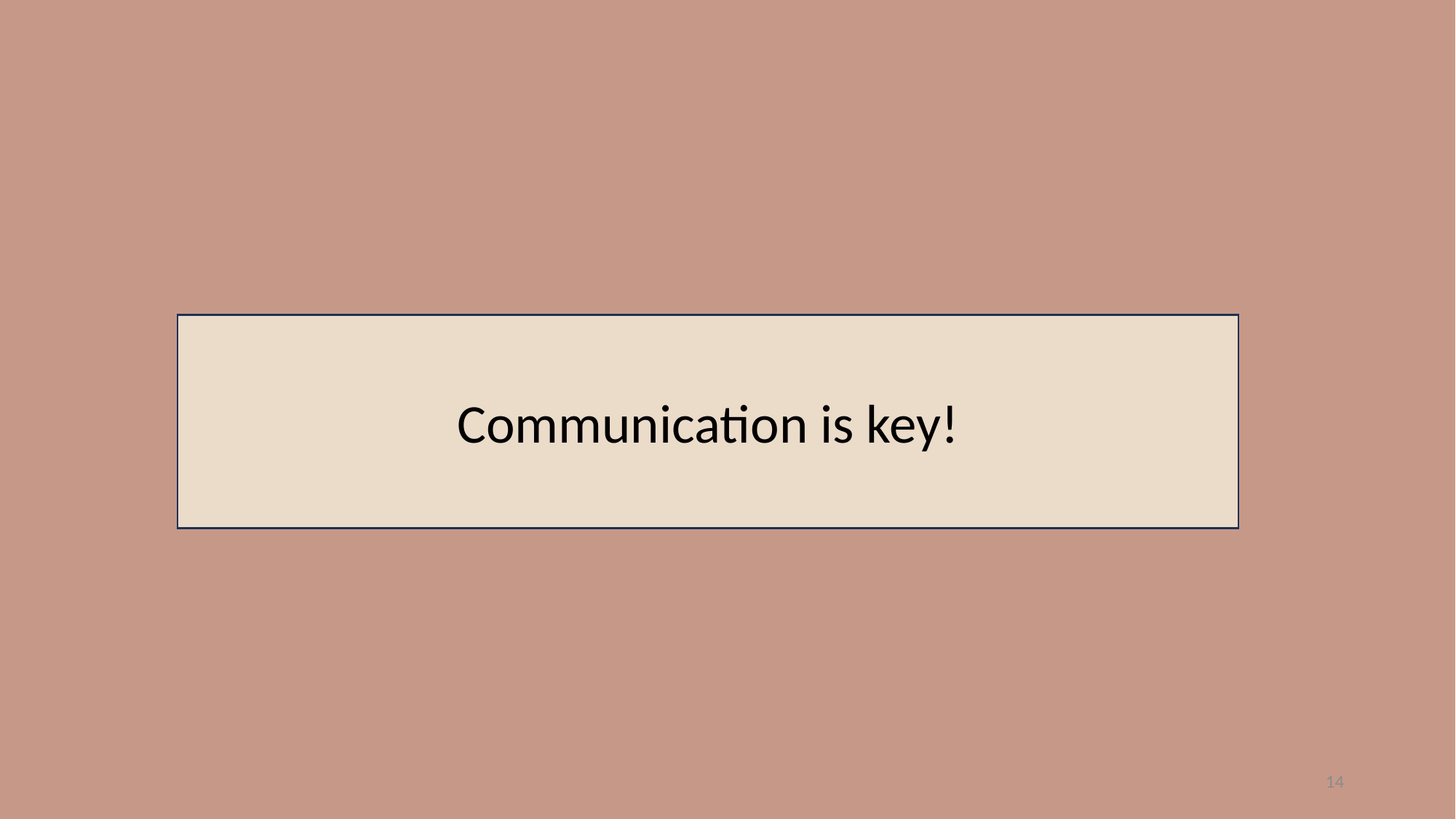

Communication is key!
14

## Slide 15
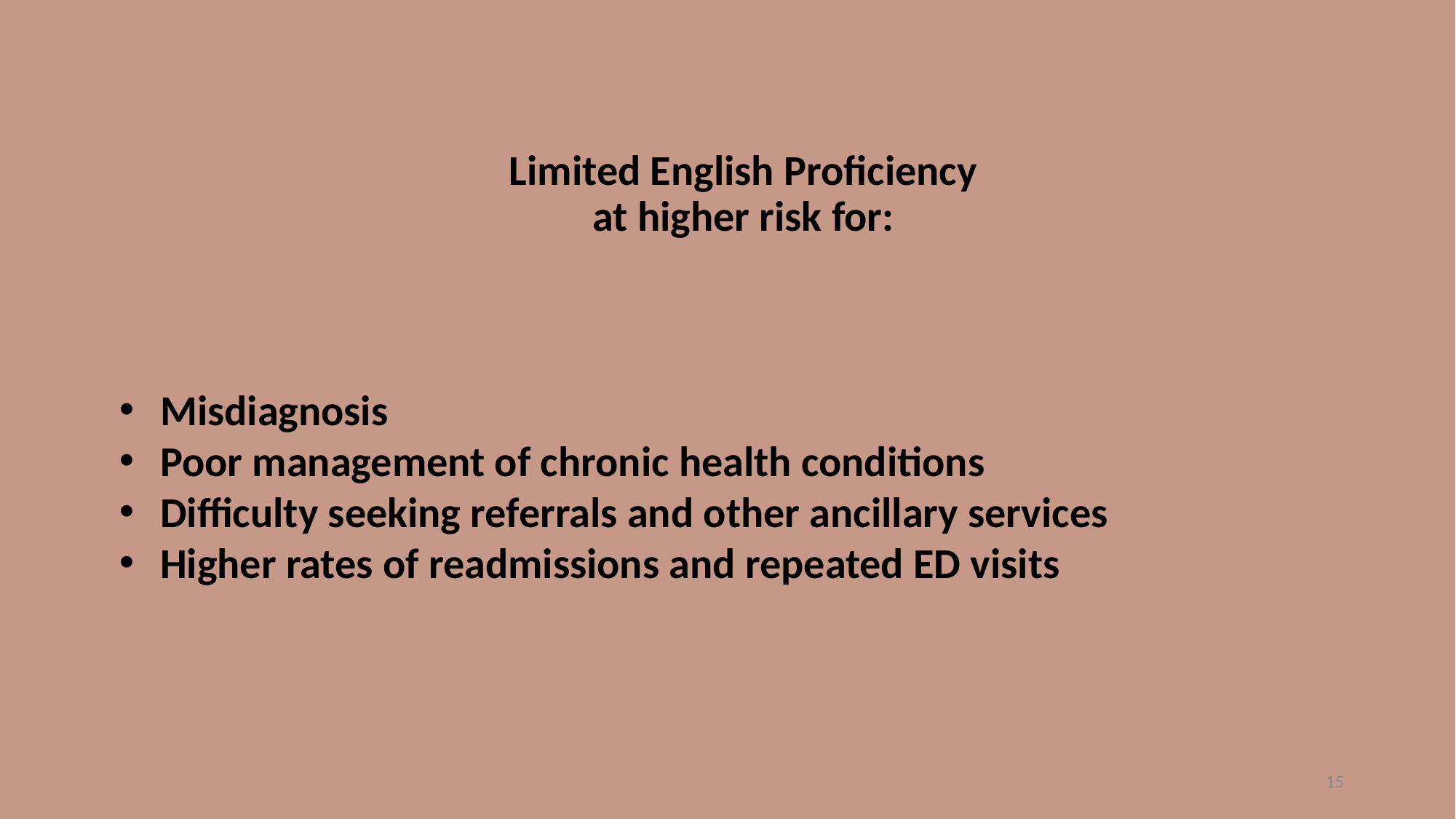

# Limited English Proficiency at higher risk for:
Misdiagnosis
Poor management of chronic health conditions
Difficulty seeking referrals and other ancillary services
Higher rates of readmissions and repeated ED visits
15

## Slide 16
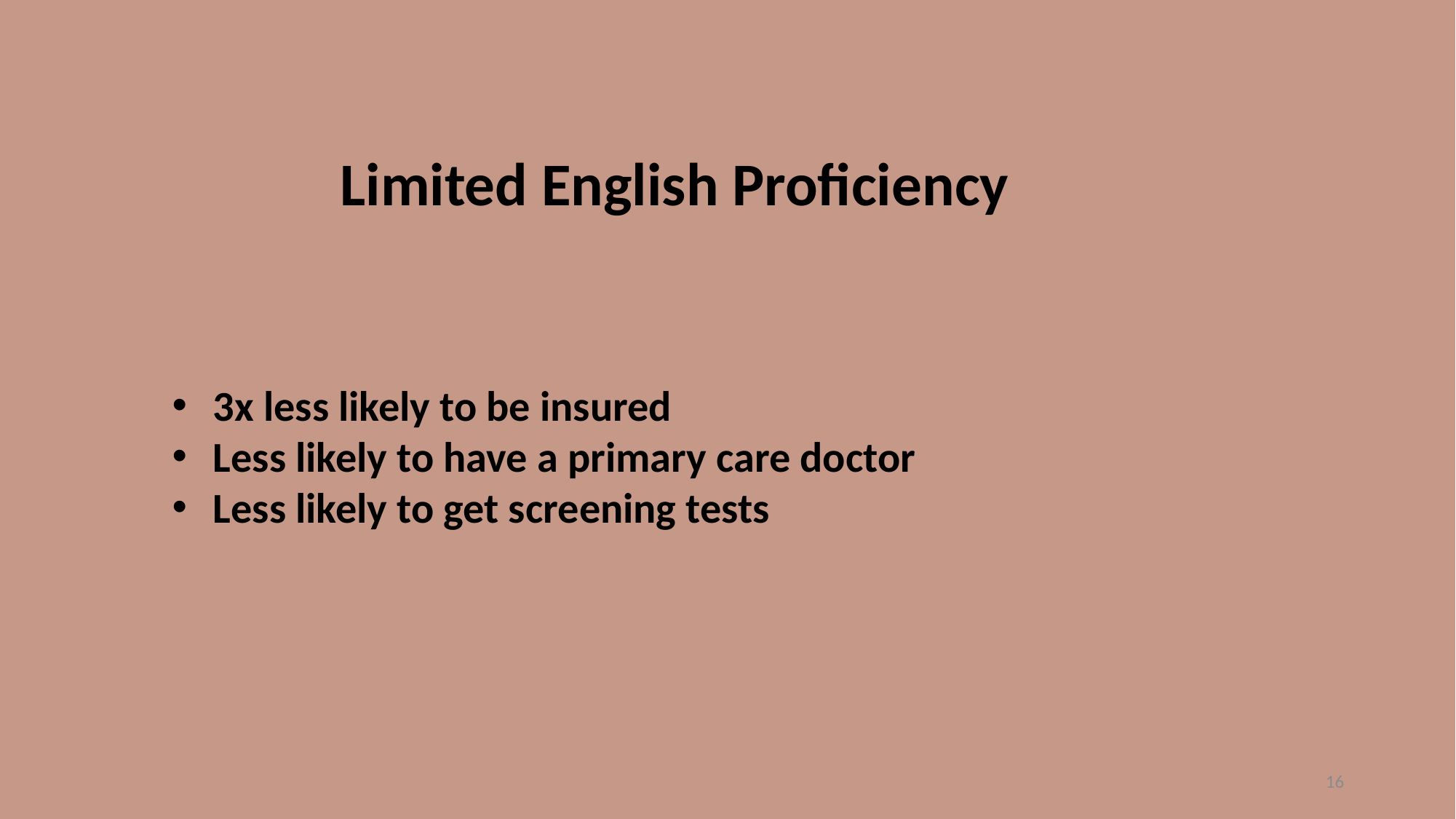

# Limited English Proficiency
3x less likely to be insured
Less likely to have a primary care doctor
Less likely to get screening tests
16

## Slide 17
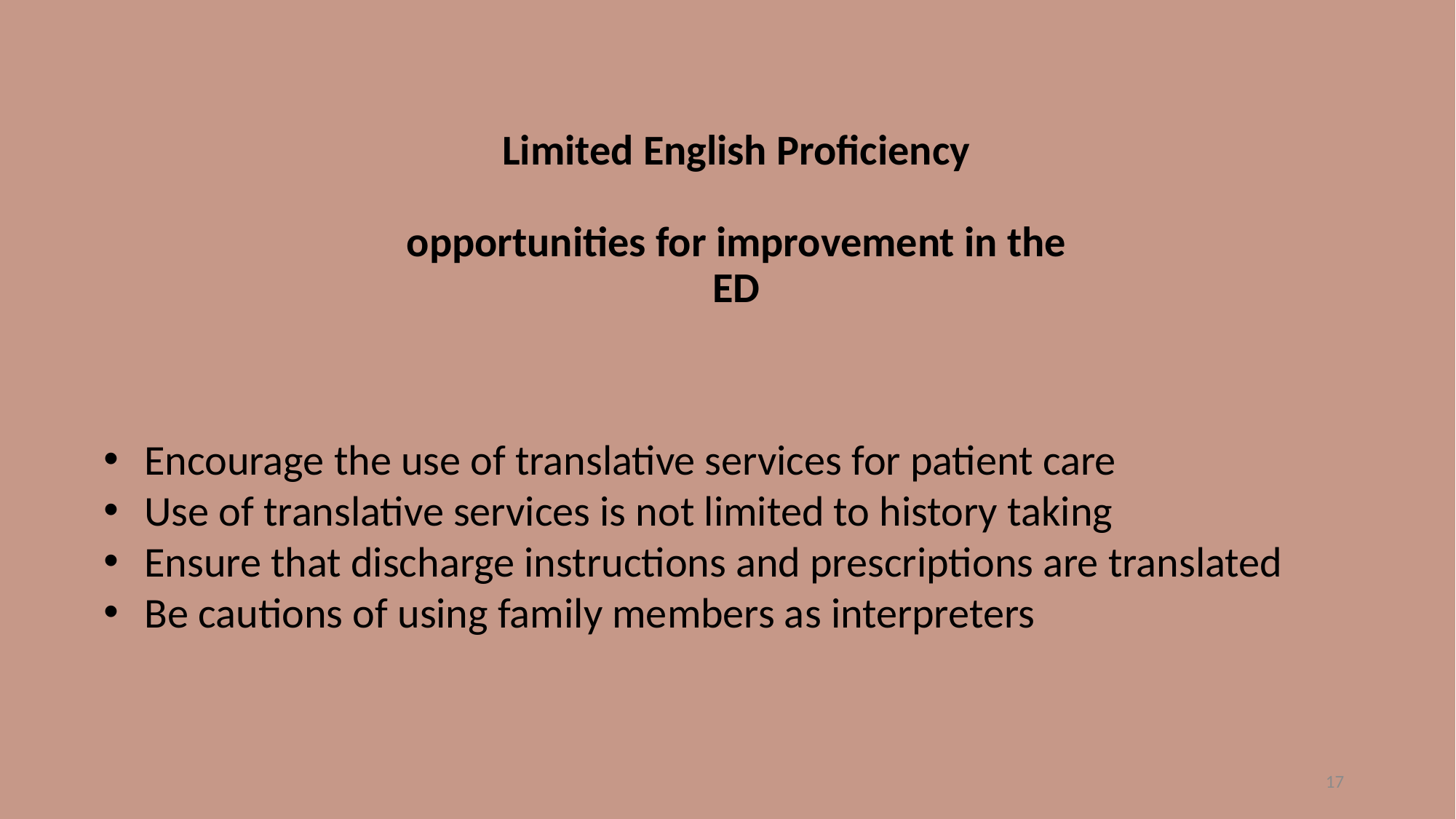

# Limited English Proficiency opportunities for improvement in the ED
Encourage the use of translative services for patient care
Use of translative services is not limited to history taking
Ensure that discharge instructions and prescriptions are translated
Be cautions of using family members as interpreters
17

## Slide 18
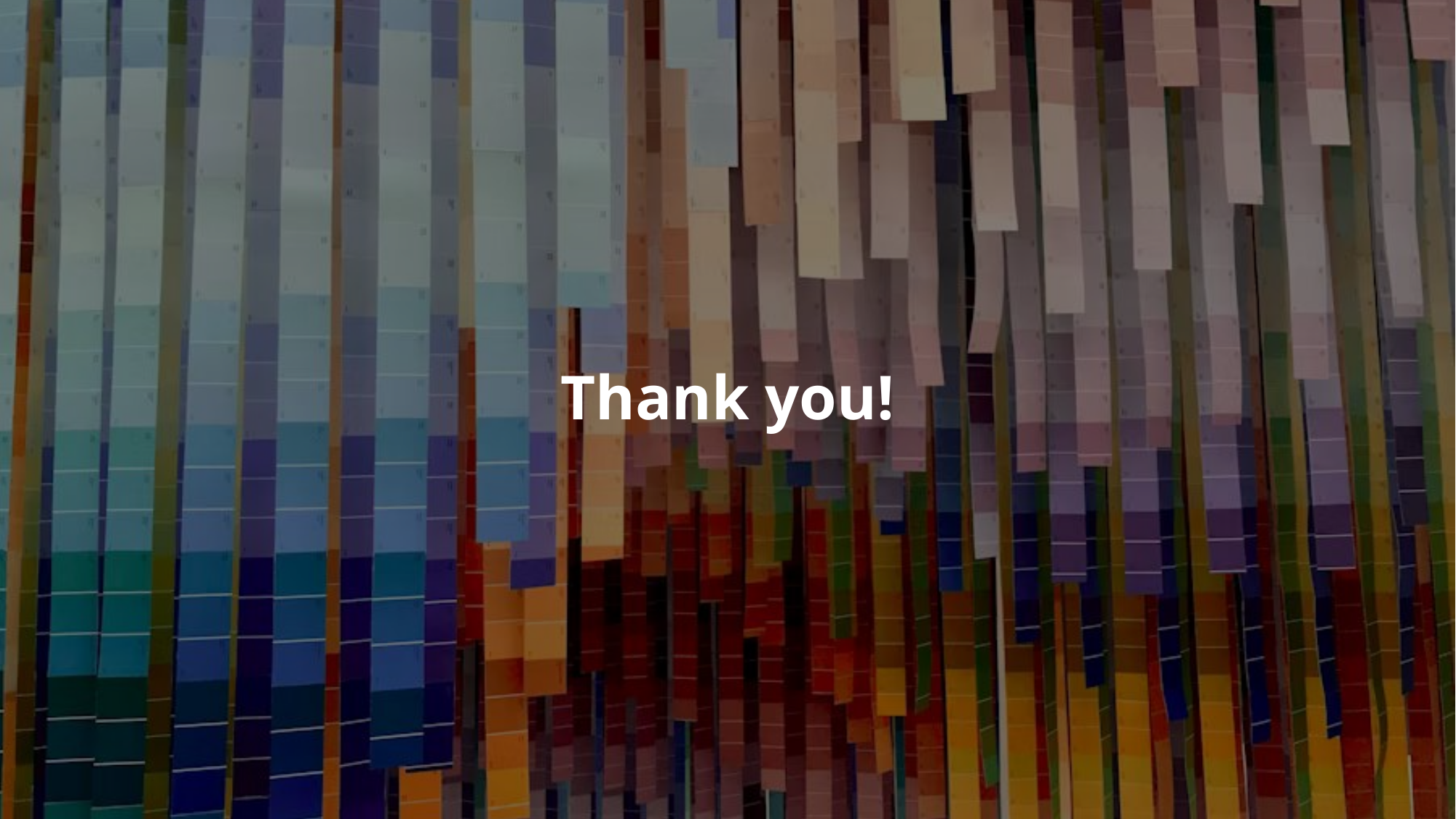

# Thank you!
18
